# Mitochondrial-Targeted Therapy for Doxorubicin-Induced Cardiotoxicity

**DOI:** 10.3390/ijms23031912

**Published:** 2022-02-09

**Authors:** Bin Bin Wu, Kam Tong Leung, Ellen Ngar-Yun Poon

**Affiliations:** 1 Centre for Cardiovascular Genomics and Medicine, Lui Che Woo Institute of Innovative Medicine, The Chinese University of Hong Kong (CUHK), Hong Kong SAR, China; binbinwu@cuhk.edu.hk; 2 Hong Kong Hub of Paediatric Excellence (HK HOPE), The Chinese University of Hong Kong (CUHK), Hong Kong SAR, China; ktleung@cuhk.edu.hk; 3 Department of Paediatrics, The Chinese University of Hong Kong (CUHK), Hong Kong SAR, China; 4 Department of Medicine and Therapeutics, The Chinese University of Hong Kong (CUHK), Hong Kong SAR, China; 5 School of Biomedical Sciences, The Chinese University of Hong Kong (CUHK), Hong Kong SAR, China

**Keywords:** doxorubicin (DOX), cardiotoxicity, mitochondria, human pluripotent stem cells (hPSC), anthracyclines, hPSC-cardiomyocytes

## Abstract

Anthracyclines, such as doxorubicin, are effective chemotherapeutic agents for the treatment of cancer, but their clinical use is associated with severe and potentially life-threatening cardiotoxicity. Despite decades of research, treatment options remain limited. The mitochondria is commonly considered to be the main target of doxorubicin and mitochondrial dysfunction is the hallmark of doxorubicin-induced cardiotoxicity. Here, we review the pathogenic mechanisms of doxorubicin-induced cardiotoxicity and present an update on cardioprotective strategies for this disorder. Specifically, we focus on strategies that can protect the mitochondria and cover different therapeutic modalities encompassing small molecules, post-transcriptional regulators, and mitochondrial transfer. We also discuss the shortcomings of existing models of doxorubicin-induced cardiotoxicity and explore advances in the use of human pluripotent stem cell derived cardiomyocytes as a platform to facilitate the identification of novel treatments against this disorder.

## 1. Introduction

Advances in cancer treatment have greatly improved the survival of cancer patients. For instance, children diagnosed with a malignancy before 15 years of age have a 5-year survival rate of approximately 80% [1,2]. However, the relatively high survival rate of patients is associated with a high incidence of treatment-related disorders. Anthracyclines, such as doxorubicin (DOX), are effective chemotherapeutic agents for the treatment of cancer, but their clinical use is associated with severe and potentially life-threatening cardiotoxicity [3]. Cancer survivors treated with anthracyclines are at much increased risk of cardiovascular disorders. Despite decades of research, treatment options remain limited. Dexrazoxane is currently the only drug approved by the U.S. Food and Drug Administration (FDA) for the prevention of DOX-induced cardiotoxicity [4], but it has been associated with a reduced tumor response rate to DOX and possible risk of secondary malignancies [5], greatly limiting its use. Considering the widespread use of anthracyclines in general, and DOX in particular, alternative treatment options are urgently needed. Here, we review the pathogenic mechanisms of DOX-induced cardiotoxicity, including both established (e.g., oxidative damage) and recently discovered (e.g., ferroptosis) mechanisms. We also present an update on therapeutic strategies against this disorder, with a focus on those that can target and/or protect the mitochondria and cover different therapeutic modalities encompassing small molecules, post-transcriptional regulators, and mitochondrial transfer. We reason that the inadequacies of existing models of DOX-induced cardiotoxicity might contribute to the inability to translate findings in animal studies to the clinic; thus, we will discuss the shortcomings of rodent models commonly used to study this disorder and explore recent advances in human pluripotent stem cell technology to facilitate investigations of disease pathogenesis and the identification of novel treatment against this disorder.

## 2. Types of Anthracyclines

**Figure 1:**
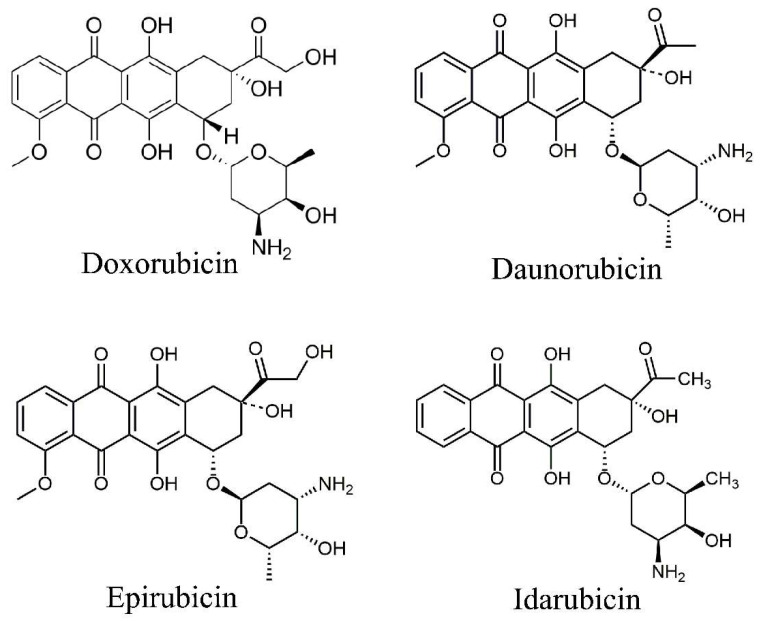
The chemical structure of anthracyclines.

DOX, daunorubicin, epirubicin, and idarubicin are the main anthracyclines approved by the FDA for clinical use. Among them, DOX and daunorubicin were isolated from *Streptomyces peucetius* var. *caesius* in the early 1960s, while epirubicin and idarubicin are synthetic analogues of DOX and daunorubicin (Figure 1). Structurally, all anthracyclines contain an anthraquinone ring system (aglycone), but specific structural differences (such as the presence of methyl or alcohol groups) confer them with different spectrums of anticancer activities. DOX is the most commonly used chemotherapeutic treatment and is effective against many types of cancers, including both solid and non-solid malignancies, while daunorubicin is often used to treat acute lymphoblastic and myeloblastic leukemias [6].

The use of anthracyclines is associated with significant adverse effects, such as cardiotoxicity, nausea, and vomiting, the most serious of which is cardiotoxicity. The anthracyclines differ in their cardiotoxic potential, with DOX being the most cardiotoxic (but also considered to be the most effective against different tumors), while epirubicin is considered the least damaging. Consequently, we will focus our review on DOX, whose cardiotoxicity is the most significant and well-characterized.

## 3. Clinical Features of DOX-Induced Cardiotoxicity

DOX has been widely reported to induce dose-dependent, progressive, and potentially lethal myocardial damage. Toxicity is usually apparent within one year of treatment in the form of reduced left ventricular ejection fraction (LVEF), although late onset toxicity in patients, such as in children, are occasionally reported. Cardiomyopathy may progress to congestive heart failure [6,7]. Once this develops, ~50% of patients die within 2 years [8]. The relationship between congestive heart failure and the cumulative dose of anthracyclines and DOX has been well documented. Mitry et al. reported a 4% risk of heart failure when the cumulative dose is under 500 mg/m^2^, and this risk increases to 36% when the cumulative dose is more than 600 mg/m^2^ [9]. In a retrospective study, the incidence of heart failure was found to be 5% at a cumulative dose of 400 mg/m^2^, 16% at a dose of 500 mg/m^2^, 26% at a dose of 550 mg/m^2^, and rose to 48% when the dose reached 700 mg/m^2^ [10]. Another study by Lefrak et al. indicated that the incidence rate of congestive heart failure was only 0.27% in patients who were treated with less than 550 mg/m^2^ of DOX, and 30% in patients who received dose more than 550 mg/m^2^ [11]. Based on these results, the recommended maximum life-time cumulative dose of DOX is 400–450 mg/m^2^ due to higher risk of adverse cardiac events above this level [12]. However, it should be noted that doses of anthracyclines below this threshold may still pose a significant risk of cardiotoxicity. A recent study by Khanna et al. showed that exposure to ≥250 mg/m^2^ of DOX equivalent anthracycline chemotherapy is a statistically significant predictor of heart failure among survivors of childhood cancers (hazard ratio 8.6) [13]. Even anthracycline doses of ≤250 mg/m^2^ have been shown to induce cardiac abnormalities [14,15]. Leger et al. reported that a proportion of childhood cancer survivors exposed to a very low anthracycline dose of ≤100 mg/m^2^ demonstrated subclinically abnormal left ventricular structure [15]. Although it is yet unclear whether such abnormalities would progress to clinically significant disease, further investigations are needed to reveal the long-term consequences of low dose anthracycline treatment.

Total cumulative dose, as well as very young and very old age, are established risk factors for anthracycline-induced cardiotoxicity. However, the role of gender as a risk factor is not well defined. There is much evidence to show that the female gender is at an increased risk of anthracycline-induced cardiotoxicity among the pediatric population, and this is comprehensively reviewed by Meiners et al. [16]. By contrast, the link between gender and risk is less established among adult patients, with some studies in fact suggesting that the male gender is more at risk of adverse cardiac events [17]. Preclinical studies in mice also manifest sexual dimorphism in the development of DOX-induced cardiotoxicity. Zeiss et al. compared the responses of age-matched male and female mice to 25 mg/kg DOX, and found, in general, greater severity in males [18].

The adverse effects of anthracyclines are reported to be related to the maximum plasma concentration (Cmax) administered, while the antitumor effect of these agents is associated with the area under the plasma concentration time curve (AUC) [19]. Thus, reducing the Cmax of DOX treatment represents a potential way to alleviate toxicity. Indeed, a 10-fold decrease of Cmax without a decrease of AUC was observed when DOX was administered as a 4-h infusion as compared to an intravenous bolus injection, and this is associated with reduced cardiotoxicity [19]. Bolus DOX treatment with a median dose of 420 mg/m^2^ induced cardiotoxicity in 62% of patients, while continuous infusion with a higher median dose of 540 mg/m^2^ induced toxicity in 42% of patients (age ≥ 16) [20]. Consistent with this, the incidence of cardiotoxicity in adults is lower with a low-dose, multiple-weekly regimen compared to a single, high-dose administration [21]. However, contrary to the above, continuous DOX infusion in childhood cancer patients (e.g., acute lymphoblastic leukemia) did not confer a cardioprotective advantage compared to bolus therapy [22,23]. Furthermore, long-term survivors of childhood acute lymphoblastic leukemia treated with lower cumulative doses of DOX (median dose 352 mg/m^2^) were found to experience late-onset cardiac complications [24]. Therefore, although altering the dosing and administration of treatment may partially alleviate the cardiotoxicity of DOX, better cardioprotective strategies are needed.

## 4. The Mechanisms of DOX-Induced Cardiotoxicity

**Figure 2:**
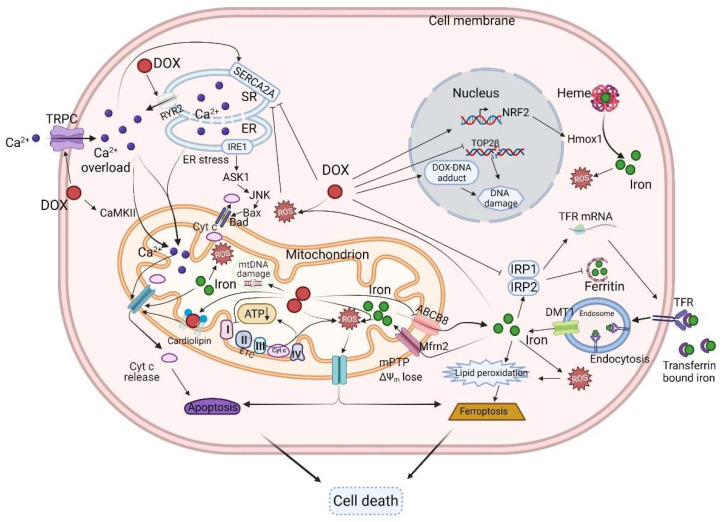
Schematic diagram of the mechanisms of DOX-induced cardiotoxicity. DOX can suppress the function of the ETC, and thereby reduce ATP levels. Increased ROS production induces the opening of the mPTP and the release of pro-apoptotic proteins such as cyt C. DOX can also induce calcium overload by altering the levels and activities of Ca^2+^ handling proteins such as TRPC, RYR2, and SERCA2A. DOX can disturb iron uptake and storage, leading to iron overload, apoptosis, and ferroptosis. DOX can complex with topoisomerase to induce DNA damage. DOX, doxorubicin; ER, endoplasmic reticulum; TRPC, transient receptor potential canonical; IRP, iron-regulatory protein; TfR, transferrin receptor; DMT1, divalent metal transporter 1; mPTP, mitochondrial permeability transition pore; Δψm, mitochondrial membrane potential; TOP2β, topoisomerase 2β; RYR2, ryanodine receptor 2; SERCA2A, sarcoplasmic/endoplasmic reticulum calcium ATPase 2; Mfrn2, mitoferrin-2; ABCB8, ABC protein B8; ETC, electron transport chain; Cyt C, cytochrome C. Created with BioRender.com (accessed on 28 December 2021).

Despite decades of research, the molecular mechanisms of DOX-induced cardiotoxicity remain controversial, with various contradictions between experimental and clinical data. Therefore, the cardiotoxic mechanisms of DOX are likely to be complex and multi-factorial (Figure 2).

### 4.1. The Mitochondria

One of the most studied targets of DOX is the mitochondria. The mitochondria are critical for energy production. As the most metabolically active organ in the body, the heart possesses the highest mitochondrial content of all tissues. The mitochondria comprise ~30% of the volume of adult cardiomyocytes, and are responsible for producing ~90% of the ATP in these cells via oxidative phosphorylation [25]. Cardiomyocytes primarily utilize fatty acids as fuel, and ATP is generated by the electron transport chain, a series of five complexes (complex I–V and the F1FO-ATP synthase) located in the inner mitochondrial membrane [26]. Furthermore, the mitochondria also regulate critical biological processes. The electron transport chain, particularly complexes I (NADH dehydrogenase), II (succinate oxidoreductase), and III (cytochrome c reductase), is an important producer of ROS [27], and the mitochondria can mediate apoptosis. Damage to this organelle can therefore severely impair cardiac function.

### 4.2. DOX Accumulates in the Mitochondria and Perturbs Mitochondrial Bioenergetics and Function

DOX is cationic and has hydrophilic and hydrophobic regions which allow it to freely penetrate the membranes of cytoplasmic organelles. It has been reported that DOX accumulates in the mitochondria at concentrations 100-fold higher than in plasma [8]. Thus, the mitochondria are commonly considered to be a major target of DOX toxicity. Consistent with this, the disruption of cardiac bioenergetics is a hallmark of DOX-induced cardiotoxicity. Cytosolic and mitochondrial creatine kinase (CK), creatine (Cr), and phosphocreatine (PCr) are important components of the cellular ATP buffer and energy transport system. DOX can disrupt intracellular ATP pools by impairing the structure and function of mitochondrial CK, an enzyme which catalyzes the reaction of Cr to PCr (ATP pool replenisher), resulting in the reduction of ATP levels [8,28].

DOX has been reported to inhibit components of the electron transport chain via multiple mechanisms [29]. For instance, Marcillat et al. found that redox recycling of DOX can significantly and severely inhibit the activity of mitochondrial complex I [30]. During forward electron transport, DOX acts as a one-electron acceptor, accepting one electron from NADH dehydrogenase of complex I at the expense of NADH [31]. The inhibition of the mitochondrial complex I triggers the opening of the mitochondrial permeability transition pore (mPTP), leading to apoptosis [32]. Another target of DOX is cytochrome c oxidase, the terminal enzyme in the mitochondrial respiratory chain. DOX inhibits the activity of cytochrome c oxidase in vitro and in vivo [30,33,34,35]. The expression of COX5A, a subunit of cytochrome C oxidase, was found to be significantly reduced in cardiomyocytes after DOX treatment. Overexpression of COX5A could attenuate DOX-induced apoptosis, as well as preserve mitochondrial respiration and mitochondrial membrane potential [36].

DOX has also been shown to perturb mitochondrial function by inhibiting members of the sirtuin family, which catalyse the deacetylation of histone and non-histone lysine residues. DOX can inhibit SIRT3, also known as mitochondrial sirtuin, a mitochondrial NAD^+^-dependent protein deacetylase [37]. SIRT3 plays an important role in regulating mitochondrial bioenergetics. SIRT3 maintains oxidative phosphorylation via promoting fatty acid oxidation and pyruvate utilization [38]. It also regulates ROS generation at the electron transport chain by increasing the expression/activities of antioxidant enzymes [39,40] and maintains mitochondrial dynamics by targeting OPA1 [41,42]. DOX treatment can suppress SIRT3 expression in a mouse heart, as well as H9c2 and primary cardiomyocytes [43,44]. The overexpression of SIRT3 attenuates ROS levels, preserves mitochondrial function, and protects mitochondrial DNA from damage upon DOX treatment [39,44]. SIRT1, another sirtuin family member, is expressed widely in tissues, and is located in both the nucleus and cytoplasm of adult cardiomyocytes [45]. By deacetylating histone and non-histone proteins, SIRT1 acts as a multifunctional protein that regulates various biological processes, including heart development, autophagy, apoptosis, circadian clock, and lifespan [46], as well as cardiac electrical activity [47]; further, it can protect the heart against myocardial ischemia/reperfusion injury [48]. DOX inhibits the expression of SIRT1. SIRT1 deficiency aggravates DOX-induced cytotoxicity and disrupts mitochondrial biogenesis and function [49,50]. Conversely, SIRT1 inhibits DOX-induced mitochondrial dysfunction and cell death [51], and the overexpression of SIRT1 suppresses DOX-induced apoptosis and ROS production [52]. In addition, SIRT1 can deacetylate PGC-1α, activating a suite of genes [50] involved in mitochondrial function and biogenesis, such as nuclear respiratory factor-1 (*Nrf1*) [49,53]. Consistent with this, a recent study showed that NRF1 could protect human-induced pluripotent stem cells (hiPSC)-derived cardiomyocytes against a range of cardiotoxins, including DOX [54]. In summary, sirtuins can regulate mitochondrial function and thereby promote cardiomyocyte survival. These experiments established sirtuins as potential therapeutic targets for DOX-induced cardiotoxicity.

DOX has been shown to damage the mitochondria by irreversibly binding with, and inhibiting, cardiolipin. Cardiolipin is a phospholipid in the mitochondrial inner membrane which is required for the activities of respiratory chain enzymes including cytochrome c oxidase and cytochrome c oxidoreductase [55,56]. The daunosamine sugar moiety of DOX possesses a positive charge at physiological pH, which endows it with a high affinity for cardiolipin [57]. The binding of DOX to cardiolipin renders the latter incapable of acting as a cofactor for mitochondrial respiratory enzymes. Additionally, cardiolipin serves as an anchor for cytochrome c, with about 15% protein of cytochrome c being tightly bound to cardiolipin [58]. The formation of the irreversible DOX–cardiolipin complex reduces the availability of cardiolipin to anchor cytochrome c, leading to the release of cytochrome c from the mitochondria and, consequently, the initiation of apoptosis [59].

Most mitochondrial proteins are encoded by nuclear DNA and then imported into the mitochondria [60]. Phospholipids such as cardiolipin, phosphatidylethanolamine (PE), and phosphatidylserine (PS) are important for mitochondrial protein import [60]. DOX can interact with phospholipids to modify mitochondrial membrane properties and disrupt transporter activities [60,61,62,63]. In addition to the aforementioned studies about the effect of DOX on cardiolipin, Bellance et al. reported that DOX could inhibit PS decarboxylase and alter the PS/PE ratio in the mitochondrial membrane, modifying the mitochondrial membrane composition [64]. Consistent with this, another study showed that DOX can act as an effective inhibitor of the translocation-dependent decarboxylation of PS to PE and disrupt the transport of PS between the outer and inner mitochondrial membrane [65]. Therefore, by interacting with phospholipids, DOX may disrupt the import of proteins into the mitochondria, which may in turn contribute to the cardiotoxicity of DOX.

### 4.3. ROS and DOX-Induced Cardiotoxicity

**Figure 3:**
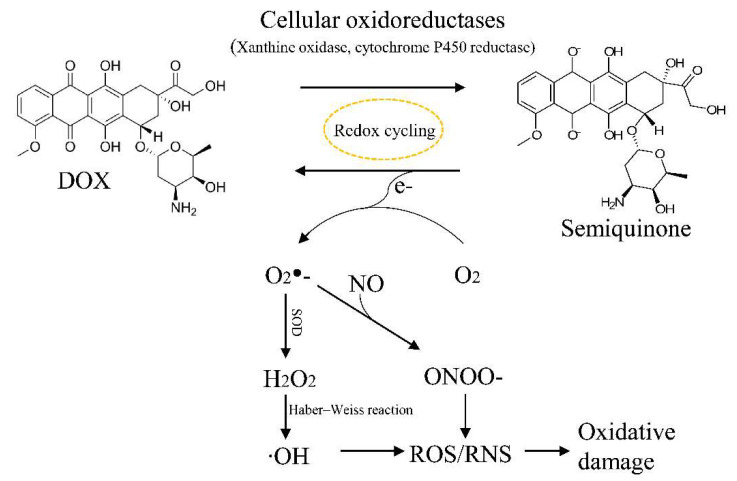
Schematic diagram of DOX-induced intracellular ROS generation. DOX was reduced to semiquinone by cellular oxidoreductases, and then parent quinone was regenerated by transferring an electron to oxygen (O_2_). The resulting superoxide (O_2_^−^) radical initiates the formation of ROS. NO, nitric oxide; RNS, reactive nitrogen species; SOD, superoxide dismutase.

Oxidative stress has long been considered to be a pathogenic contributor to DOX-induced cardiotoxicity. DOX can promote the generation of ROS through multiple mechanisms (Figure 3). DOX can be reduced by mitochondrial complex I or by cytochrome P450 reductase to generate the semiquinone radical, which can then react with molecular oxygen to be re-oxidized into the parent DOX molecule in a process called redox cycling [28,66]. This process generates different ROS species such as the superoxide anion radical O_2_^−^ and hydrogen peroxide (H_2_O_2_). O_2_^−^ and H_2_O_2_ can be further converted to the highly reactive and toxic hydroxyl radicals (•OH) via the Fenton and Harber–Weiss reactions [67]. Alternatively, O_2_^−^ can combine with nitric oxide to form peroxynitrite (ONOO-), which is highly potent and cytotoxic, and can damage the heart via nitrosative stress [68,69]. While moderate levels of ROS play an essential role in regulating cell proliferation and survival, high levels of ROS can lead to the oxidation of proteins, lipids, and signaling molecules, resulting in severe cellular damage [66,70].

ROS is usually maintained at a low level by the antioxidant system. However, antioxidant enzymes such as catalase and glutathione peroxidase are less abundant in cardiomyocytes than in other cell types, rendering cardiomyocytes more sensitive to oxidative stress. Furthermore, DOX has been shown to exacerbate oxidative damage by altering the levels of enzymes involved in ROS production and the antioxidant response. DOX was found to increase the mRNA expression and enzymatic activity of xanthine oxidase, an enzyme in the endoplasmic reticulum (ER) involved in the reduction of DOX to semiquinone to promote redox cycling [71,72]. DOX can also upregulate nitric oxide synthase to increase the level of nitric oxide, thereby enhancing the production of the highly toxic peroxynitrite [68]. Conversely, enzymes involved in antioxidant defense such as glutathione peroxidase 4 have been shown to be downregulated by DOX [73].

Experiments in animal models support the role of oxidative stress in DOX-induced cardiomyopathy. For instance, the transgenic overexpression of antioxidants, such as catalase, superoxide dismutase, thioredoxin-1, and metallothionein, could protect against DOX-induced cardiac injury [74,75,76,77], while glutathione peroxidase-1 deficient mice were susceptible to this disorder [78]. Furthermore, a range of small molecule antioxidants, including ascorbic acid and N-acetylcysteine, could ameliorate cardiotoxicity, showing that oxidative stress is an important cause of DOX-induced cardiotoxicity [9,79,80].

### 4.4. Iron, Ferroptosis, and DOX-Induced Cardiotoxicity

Iron is critical for a broad range of biological processes such as energy metabolism and cellular respiration. However, excess iron or iron overload can lead to toxicity. Hemochromatosis, which is associated with excessive iron accumulation, can lead to heart failure [81]. Animal models of iron overload, such as mice deficient of the HFE protein, are more susceptible to cardiac injury post DOX treatment, demonstrating a link between iron metabolism and DOX-induced cardiomyopathy [82].

Iron is present in the plasma in a soluble form bound to transferrin. Transferrin-bound iron combines with the transferrin receptor, forming a transferrin-dimeric transferrin receptor (TfR) complex, which allows iron to pass through the cell membrane via endocytosis. Iron in the form of ferrous ion (Fe^2+^) can also be transported into the cytosol via divalent metal transporter 1 (DMT1). Once inside the cell, iron becomes part of the labile iron pool, which can be incorporated in important cellular enzymes such as heme and iron–sulfur clusters, or be sequestered by the iron storage protein ferritin [83].

**Figure 4:**
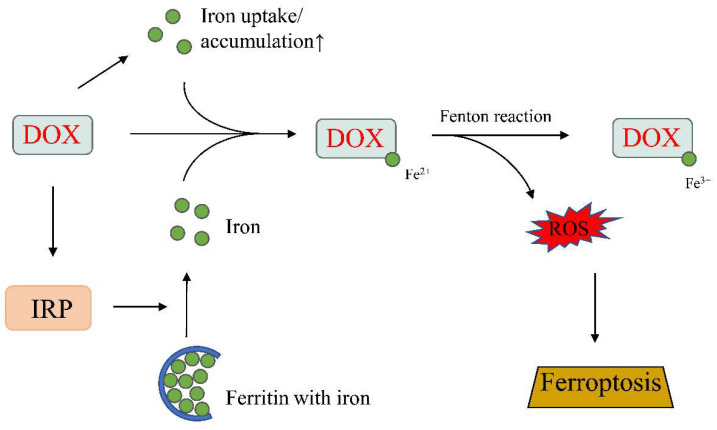
DOX-induced ferroptosis. DOX promotes iron uptake and accumulation, increases ROS production, and induces ferroptosis. IRP, iron-regulatory protein. Up arrow, increase. Created with BioRender.com (accessed on 28 December 2021).

Iron homeostasis is regulated by mRNA-binding molecules, as well as iron-regulatory proteins 1 and 2 (IRP1 and IRP2). IRPs can bind to the iron-response elements (IREs) of genes important for iron metabolism to act as either a translational enhancer or inhibitor. IRP can bind to the IRE of ferritin located at the 5′-untranslated region, and inhibits mRNA translation and, hence, decreases iron storage. Therefore, IRP-1 and -2 are important regulators of the labile iron pool. DOX can increase the labile iron pool by modulating the activities of IRP-1 and -2. DOX has been shown to inactivate both IRP-1 and/or IRP-2 in cardiomyocytes [84,85]. The alcohol metabolite of DOX and the DOX–Fe complex can affect the regulatory function of IRP-1, as well as disturb the transferrin-mediated iron uptake and ferritin-mediated iron storage [84,86] (Figure 4).

Iron homeostasis in the mitochondria is maintained by a number of proteins located in the mitochondrial inner membrane [87,88]. Iron entry is mediated by mitoferrin-2, while iron export is less understood but is thought to involve the ABCB7 and ABCB8 transporters [88,89]. Disturbed mitochondrial iron homeostasis is detrimental to mitochondrial function. Experiments with isolated mitochondria showed that iron overload could cause cardiac mitochondrial dysfunction by increasing ROS production, mitochondrial membrane depolarization, and mitochondrial swelling [90,91]. DOX has been shown to increase the mitochondrial iron level by downregulating ABCB8, leading to lipid peroxidation and cardiac dysfunction, while this was ameliorated in transgenic mice which overexpressed ABCB8 [89].

Iron accumulation is thought to facilitate/potentiate DOX-induced cardiotoxicity by inducing ROS production. One mechanism involves the Fenton and Haber–Weiss reactions, wherein iron promotes the formation of the highly reactive and toxic hydroxyl radical (•OH). In addition, DOX can also complex with iron to generate ROS. DOX–Fe^2+^ can react with molecular oxygen to generate superanion free radicals, which then dismutate to H_2_O_2_ and the hydroxyl free radical. Alternatively, or in addition to, the ferric ion (Fe^3+^) of DOX–Fe^3+^ can be reduced to produce a DOX free radical chelate with Fe^2+^ to generate the hydroxyl free radical [92]. Unlike the superanion free radical and H_2_O_2_, which can be de-toxified by the cells’ antioxidant defense system, the hydroxyl radical is resistant to enzymatic degradation and can modify key macromolecules essential for cellular function.

Recent reports of ferroptosis enhanced our understanding of how iron overload can lead to cardiac dysfunction. Ferroptosis is a recently discovered mode of regulated cell death characterized by iron-induced lipid peroxidation [93] and has now been linked to the pathophysiology of many cardiac disorders [73,94]. The role of ferroptosis in cardiomyopathy was reported in 2019 by Fang et al. [94]. These authors demonstrated that DOX-treated cardiomyocytes showed features typical of ferroptosis-mediated cell death, and that the inhibition of this process by ferrostatin-1 significantly suppress DOX-induced cardiomyopathy. DOX can induce ferroptosis via multiple mechanisms. Fang et al. showed that DOX could trigger ferroptosis by inducing nuclear factor erythroid 2-related factor 2 (Nrf2)-mediated upregulation of heme oxygenase-1 (Hmox1), which catalyzes heme degradation, leading to the accumulation of non-heme iron and thereby inducing toxicity [94]. Tadokoro et al. showed that DOX could induce ferroptosis in the mitochondria via the down-regulation of glutathione peroxidase 4 (GPx4). Cardiac dysfunction, in the form of reduced LVEF, was ameliorated in GPx4 transgenic mice and exacerbated in GPx4 heterodeletion mice. Experiments in cultured cardiomyocytes further demonstrated that GPx4 overexpression, or iron chelation targeting Fe^2+^ in mitochondria, prevent DOX-induced ferroptosis, revealing the mitochondria to be a main target of DOX-induced ferroptosis [73].

While much evidence in animal models supports an important role of iron accumulation in DOX-induced cardiomyopathy, attempts to protect against this disorder using iron chelators have given conflicting results. Early support for the importance of iron in DOX-induced cardiomyopathy comes from the success of dexrazoxane, an iron chelator, in animal studies and clinical trials. However, it was later realized that dexrazoxane has a broad range of activities against DOX, including antioxidant properties and the inhibition of topoisomerase [95,96]. Indeed, studies with a range of iron chelators, including deferoxamine, deferasirox [97], and Dp44mT [98], gave mixed or negative results. Along the same line, the prevention of mitochondrial-dependent ferroptosis with iron chelators could partly, but not completely, protect against cardiac damage induced by DOX [73]. Thus, iron overload is likely to be an important component of, but may not be a sole contributor to, DOX-induced cardiomyopathy.

### 4.5. Calcium Homeostasis and DOX-Induced Cardiotoxicity

Calcium is important for cardiac contraction. A set of Ca^2+^ handling proteins, including membrane channels, ATPase pumps, Ca^2+^-binding proteins, and Ca^2+^ transporters, regulate Ca^2+^ influx and efflux, thereby regulating Ca^2+^ levels within the cell [99,100,101]. Ca^2+^ can enter the cell via membrane channels such as the L-type Ca^2+^ channel and the transient receptor potential canonical (TRPC) family channels. Ca^2+^ influx then triggers the release of Ca^2+^ from intracellular stores, the sarco(endo)plasmic reticulum (SR), via the ryanodine receptors (RyRs) in a process termed “calcium-induced calcium release”. Ca^2+^ can be extruded through sodium/calcium exchangers or returned to the SR via sarcoplasmic/endoplasmic reticulum calcium ATPase 2 (SERCA2A).

DOX has been shown to perturb Ca^2+^ homeostasis by modulating the expression and activities of Ca^2+^ handling proteins. DOX can increase the expression of TRPC3 and TRPC6 in adult rat ventricular myocytes and alter Ca^2+^ transient properties, while the pharmacological inhibition of the TRPC channel can prevent the DOX-induced increase of ER stress-related protein expression and intracellular Ca^2+^ levels [102]. In addition, DOX, and its metabolite doxorubicinol, can bind to, and modify the activities of, RyR2 and SERCA2A, thereby disrupting SR Ca^2+^ handling [103]. Ca/calmodulin-dependent protein kinase II (CaMKII) is yet another contributor to DOX-induced cardiac damage [104]. DOX can increase the phosphorylation of CaMKII and promote CaMKII-dependent SR Ca^2+^ leakage, resulting in diastolic calcium overload in rat myocytes and diminished SR Ca^2+^ content. Consistent with CAMKII as a mechanistic target of DOX, the pharmacological and genetic inhibition of CaMKII could attenuate Ca^2+^ abnormalities induced by DOX [104].

Perturbed Ca^2+^ homeostasis can damage cardiac function acutely and chronically by inducing apoptosis and hypertrophy. SR Ca^2+^ leak is associated with an increased mitochondrial Ca^2+^ level and ROS production. At high mitochondrial matrix Ca^2+^ concentration, the mitochondrial permeability transition pores in the inner mitochondrial membrane opens, leading to mitochondrial membrane depolarization, matrix swelling, outer membrane rupture, and the release of apoptotic signaling molecules, such as cytochrome c, from the intermembrane space, leading to irreversible injury [105]. Sustained elevated levels of intracellular calcium can also activate calcineurin, a calcium-dependent phosphatase involved in the regulation of many processes. For instance, calcineurin can dephosphorylate and activate nuclear factor of activated T-lymphocytes (NFAT), which is a critical mediator of cardiac hypertrophy and apoptosis. DOX has been shown to induce NFAT nuclear translocation, and this was associated with the increased expression of its target gene, *FasL*, culminating in the induction of apoptosis [106].

**Figure 5:**
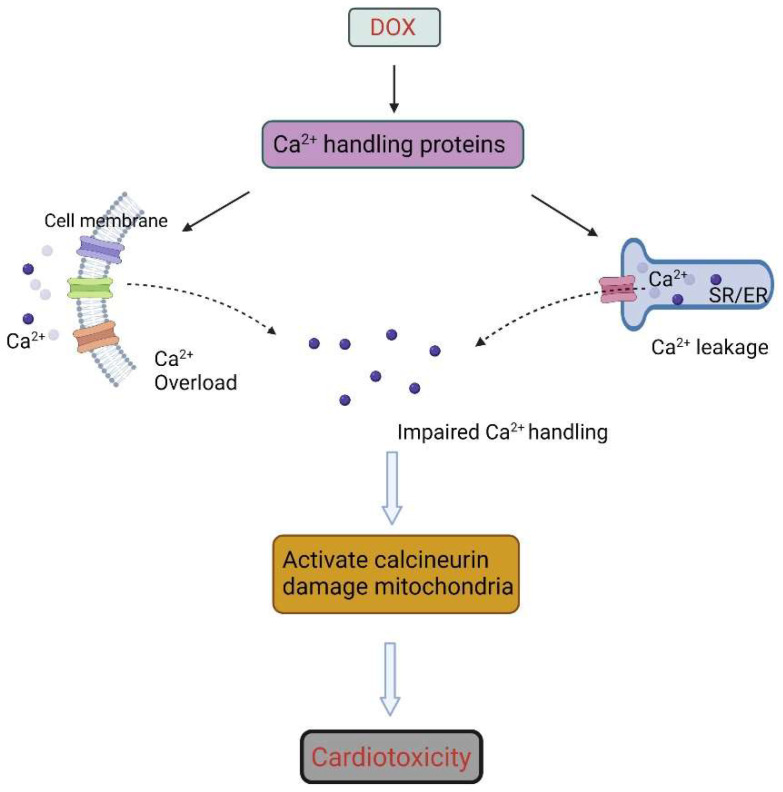
DOX can induce calcium overload by increasing calcium influx and intracellular calcium release. SR, sarcoplasmic reticulum; ER, endoplasmic reticulum. Created with BioRender.com (accessed on 28 December 2021).

Furthermore, DOX can cause calcium overload in the mitochondria by inducing ER stress [107]. The ER is an extensive intracellular membranous network involved in Ca^2+^ storage and signaling, and ER stress leads to the release of Ca^2+^ from the ER lumen, inducing the sustained accumulation of Ca^2+^ in the mitochondrial matrix and triggering mitochondrial alterations, such as permeability transition, dissipation of the electrochemical potential, matrix swelling, Bax relocalization, and the release of cytochrome c [108,109]. Furthermore, excessive ER stress may trigger the unfolded protein response (UPR) pathway. DOX has been reported to increase the UPR through the upregulation of PKR-like ER eIF2α kinase-eIF2 signaling [110] and increase the accumulation of misfolded or unfolded proteins. The latter accumulation activates the UPR transducer, inositol-requiring protein 1 alfa (IRE1α), in the ER lumen, leading to the phosphorylation of ASK1, a redox-sensor mitogen-activated protein kinase kinase kinase (MAPKKK), and inducing the activation of the JNK pathway. Ultimately, this promotes the translocation and expression of pro-apoptotic proteins, Bax and Bad, leading to the release of mitochondrial cytochrome c, apoptosis, and the development of heart failure [111,112] (Figure 5).

### 4.6. Topoisomerase and DOX-Induced Cardiotoxicity

Topoisomerases are highly conserved enzymes which regulate DNA topology. They participate in a broad range of processes, such as replication, transcription, chromatin remodeling, recombination, and repair, by facilitating transient DNA single (Topoisomerase type I) or double (Topoisomerase type IIs) strand breaks [9]. Topoisomerase II (including Top2α and Top2β) catalyzes a set of topological isomerization reactions which cleave duplex DNA via a transient protein–DNA crosslink intermediate named covalent protein-DNA Top2 cleavage complex [113,114]. Top2α, a nuclear isozyme, is overexpressed in proliferating tissues (e.g., tumor cells) and is thought to be the primary molecular target of DOX for anticancer effects [115]. DOX has been shown to interact with DNA and Top2α to form the ternary Top2–doxorubicin–DNA complex, which induces cell death. Although adult cardiomyocytes do not express a detectable level of Top2α, they express Top2β, an isozyme with similar structural features as Top2α [116] [117].

Top2β is increasingly recognized as the main target for DOX-induced cardiotoxicity based on a range of in vitro and in vivo studies. Zhang et al. demonstrated the role of Top2β in DOX-induced cardiotoxicity in a mouse model. Mice with a cardiac-specific deletion of Top2β (Top2β^∆/∆^ mice) had reduced double strand breaks and apoptosis, as well as normalized transcriptome changes associated with defective mitochondrial biogenesis, compared to the control. Importantly, the cardiac-specific deletion of Top2β protected against the development of DOX-induced heart failure, showing that Top2β is a critical mediator of DOX-induced cardiotoxicity [117]. Indeed, dexrazoxane could bind to the ATPase domain of Top2 and thereby prevent the latter from binding with DOX [118]. The blocking and degradation of Top2β is now considered the major mechanism by which dexrazoxane alleviates DOX-induced cardiotoxicity [96].

### 4.7. DNA Damage

DOX treatment has been shown to induce long-lasting effects on gene expression and DNA maintenance. For example, Berthiaume et al. reported the persistent alteration in the expression of genes associated with mitochondrial processes, glycolysis, and fatty acid oxidation in the hearts of Sprague Dawley rats after six weekly DOX injections followed by a 5-week, drug-free period [119]. While DOX is widely reported to damage nuclear DNA in cardiomyocytes, mitochondrial DNA (mtDNA) maybe more susceptible to DOX-induced damage because of its comparably low repair capacity and its proximity to the respiratory chain. Consistently, mtDNA damage, mutation, and deletion have been detected in DOX-treated mouse cardiomyocytes and in the heart of patients with DOX-induced cardiomyopathy [120,121,122,123]. Considering that mtDNA codes 37 genes necessary for the assembly of the oxidative phosphorylation machinery [124], it is not surprising that the activities of mtDNA-encoded respiratory chain enzymes (e.g., dinucleotide hydrogen dehydrogenase and cytochrome c oxidase) are diminished by DOX [123], which may be a vital factor contributing to the compromised bioenergetics induced by this agent.

## 5. Modulating the Mitochondria to Alleviate DOX-Induced Cardiotoxicity

Mitochondrial dysfunction, such as impaired bioenergetics, mitochondrial membrane potential depolarization, and increased ROS production, is a hallmark of DOX-induced cardiotoxicity, as discussed in previous sections. Thus, the mitochondria have long been considered to be a therapeutic target for this disorder. In recent years, a broad range of strategies to protect against DOX have been reported. Here, we will mainly focus on those that target mitochondria to alleviate DOX-induced cardiotoxicity (Table 1).

### 5.1. Small Molecules

Consistent with the role of the mitochondria as the target of DOX, small molecules that protect mitochondrial homeostasis and function have been shown to protect against the cardiotoxic effects of DOX. Here, we will review some recent progress in this field. For a more detailed review of small molecules targeting the mitochondria, please refer to Shi et al. [136].

Golforoush et al. showed that the inhibition of the Mitogen-activated protein kinase kinase kinase kinase-4 (MAP4K4) can alleviate DOX-induced cardiotoxicity using both rat H9c2 cells and hPSC-cardiomyocytes [125]. MAP4K4 is an upstream member of the MAPK superfamily that can regulate cell death. The same group has previously shown that inhibition of this pathway using shRNA and the small molecule inhibitor DMX-5804 can improve mitochondrial function, protect against lethal oxidative stress, and reduce cardiac ischemia reperfusion injury [137]. Golforoush et al. [125] then proceeded to show that DMX-5804 could confer similar protection against DOX in in vitro models. In terms of mitochondrial homeostasis, Liang et al. 2020 showed that liensinine, a newly identified inhibitor of mitophagy, can protect against DOX in in vitro and in vivo rodent models by inhibiting DRP1, a regulator of mitochondrial fission [126]. Along the same line, melatonin and metformin have both been shown to alleviate DOX-induced cardiac damage by preserving mitochondrial function and dynamics in rats [127]. In addition, melatonin is also a potent anti-oxidant and has been shown to activate AMPK and PGC1α, which are important regulators of metabolism and mitochondrial biogenesis, to protect against DOX [138]. The same pathway is implicated in the action of dexmedetomidin, a selective agonist of the α2-adrenergic receptor. Dexmedetomidine can inhibit the ubiquitination of PGC-1α induced by DOX, and preserves PGC-1α signaling, thereby attenuating mitochondrial dysfunction, oxidative stress, and apoptosis in vitro and in vivo [128]. Augmenting the guanylate cyclase pathway represents another potential avenue to suppress oxidative stress, improve DOX-induced cardiac dysfunction, and inhibit apoptosis in vivo and in vitro [129]. The preservation of mitochondrial respiration is also thought the underlie the cardioprotective effect of phenylalanine-butyramide, a novel synthetic derivative of butyric acid, which is a short chain fatty acid produced by the gut microbiota [130]. In addition, recent work showed that nicotinamide riboside, a precursor of NAD^+^, could prevent DOX-induced cardiotoxicity. Nicotinamide riboside was shown to elevate NAD^+^ levels, thereby reducing cardiac injury and myocardial dysfunction in DOX-treated mice. Mechanistically, nicotinamide riboside is thought to act by enhancing autolysosome clearance via NAD^+^/SIRT1 signaling [131]. Similarly, SIRT1 signaling is implicated in the cardioprotective effects of berberine. Berberine is a type of alkaloid originally extracted from a Chinese plant. It was recently shown to protect the heart from DOX-induced injury by reducing ROS production, apoptosis, and mitochondrial damage through SIRT1-mediated p66Shc suppression [50]. Now, with this expanding repertoire of potentially cardioprotective compounds, it is hoped that some of these can be translated for clinical use soon.

### 5.2. Antioxidants

Propelled by numerous reports linking oxidative stress to DOX-induced cardiotoxicity, much research has been done to investigate the use of antioxidants to alleviate toxicity, with controversial results. Animal experiments typically involve the co-application of antioxidants and/or ROS scavengers with DOX and gave generally favorable results. Vitamins C, E, and N-acetylcysteine have all been shown to suppress oxidative stress in primary cardiomyocytes and/or mouse cardiac cell lines [80]. The co-administration of antioxidants have also been shown to alleviate cardiac dysfunction in rodent models of DOX-induced cardiomyopathy in vivo [139,140].

Compared to such promising data, results from clinical trials have been disappointing [141]. N-acetylcysteine, a precursor of glutathione, can act as a scavenger of ROS and was evaluated in a randomized controlled trial by Myers et al. in 1983 [142]. Among 54 adult patients with solid tumors treated with DOX, there was no statistically significant difference in the development of heart failure among the control and patients who received N-acetylcysteine. Later studies by Jo et al. and Dresdale et al. reported similar findings [143,144], suggesting that N-acetylcysteine could not prevent or reverse cardiac dysfunction or heart failure induced by DOX. Other oxidants, such as α-tocopherol, were tested as prophylaxis against DOX-induced cardiotoxicity, and were found to confer no significant benefit by Weitzman et al. [145] and Legha et al. [146]. Mixed results were also seen in the case of carvedilol, which is a β-blocker with antioxidant properties [147,148,149].

It is unclear why there is a great discrepancy between data from animal models and that from clinical trials. Species-specific differences may play a role [150]. Alternatively, or in addition, inadequate sample size, heterogeneity in the cumulative dose of anthracyclines used, cancer type, and the very definition of, and method used for, the assessment of cardiotoxicity also makes generalization difficult [151]. The efficacy of antioxidant therapy is still unclear and awaits further study.

Yet another explanation for the poor efficacy of antioxidant therapy in the clinic may be a combination of poor uptake into the body and limited delivery to the mitochondria, where DOX accumulates and where ROS levels are the highest. A novel strategy to protect against DOX-induced oxidative stress is to target the aforementioned anti-oxidants to the mitochondria, where they are most needed [152]. To this end, a number of mitochondria-targeted antioxidants have been developed. For instance, mitoQ and mitoTempo are mitochondria-targeted mimetics of coenzyme Q and superoxide dismutase, respectively [151,153]. Both are conjugated to a lipophilic cation, the triphenylphosphonium (TPP) cation, which confers oral bioavailability and accumulation in the mitochondria as driven by the mitochondrial membrane potential of the cell. Similarly, SKQ1, another mitochondria-targeted mimetic of coenzyme Q, can also be directed to the mitochondria via a similar strategy. All three compounds have demonstrated the ability to ameliorate the pathological phenotype of a range of diseases involving oxidative stress and can protect against DOX in animal models [154,155,156]. Clinical trials are ongoing to test if they confer similar benefits in patients.

### 5.3. microRNAs (miRs)

microRNAs (miRs) are non-coding RNAs with ∼22 nucleotides that act as negative transcriptional regulators through degradation or through inhibition by RNA interference, and they have been established as critical regulators of cardiac differentiation, maturation, and function [157,158]. Given the important role of miRs in post-transcriptional regulation, miRs also play an important role in DOX-induced cardiotoxicity. Various miRs have been shown to have cardioprotective properties. Pan et al. showed that the overexpression of miR146a in AC16 cells protected against DOX-induced damage, while the inhibition of this miR exacerbated it. Consistent with this, miR-146a knockout mice showed more severe cardiac dysfunction upon DOX treatment than the control. miR-146a was shown to target TATA-binding protein (TBP) associated factor 9b (TAF9b), which is a coactivator and stabilizer of p53. The inhibition of miR-146 increased TAF9b levels and increased the stability of p53, resulting in apoptosis and disturbed autophagy [132]. miR-29b was downregulated in rat myocardium upon DOX exposure, and the restoration of miR-29b improved the cardiac function of DOX-treated rats. Mechanistically, miR-29b could directly target the 3′ untranslated region of Bax to prevent apoptosis [133]. miR-378, another miRNA with the ability to enhance cell viability and inhibit cardiomyocyte apoptosis, was found to be down-regulated in the hearts of DOX-treated rats. The overexpression of miR-378 protected against DOX-induced energy imbalance and apoptosis, targeting lactate dehydrogenase A and cyclophilin A [134], a novel mitochondrial factor with antiapoptotic properties [159]. Unlike the miRs described above, miRs have also been shown to participate in the pathogenesis of DOX-induced cardiotoxicity and/or exacerbate injury. For example, Wang et al. showed that DOX treatment elevated the expression of miR-140-5p in H9c2 cells, leading to oxidative damage by targeting *Nrf2* and *Sirt2* [135]. In addition, DOX could also upregulate the expression of miR-22 in the murine heart and miR-22 antagomir inhibited DOX-induced cardiotoxicity [160]. The inhibition of miR-22 alleviated DOX-induced cardiac fibrosis and cardiac dysfunction, and mitigated mitochondrial dysfunction through the SIRT1/PGC-1α pathway. Conversely, knocking out miR-22 enhanced mitochondrial biogenesis [161]. Thus far, the investigations of miRs to alleviate DOX-induced cardiotoxicity remain pre-clinical, but it is hoped that miR mimetics and antagomirs may be used to combat this disease in patients in the future.

### 5.4. Mitochondrial Transplantation

Since mitochondrial damage, in its various forms, is strongly implicated in the pathogenesis of DOX-induced cardiac injury, one approach to address this is to replenish the mitochondria. Mitochondria can transfer between cells by tunneling nanotubes, extracellular vesicles, naked mitochondria extrusion, etc [162], to improve aerobic respiration in mammalian cell cultures and to protect against injury in animal models [163]. Therefore, the transport of mitochondria from a healthy cell can potentially replace damaged mitochondria to rescue mitochondrial function [164,165]. Consistent with this, hPSC-cardiomyocytes have been shown to take up exogenous mitochondria and incorporate them into the existing mitochondrial network, resulting in improved contractility, calcium flux, and ATP production when challenged with DOX [166,167]. The transplantation of stem cells, including mesenchymal stem cells (MSCs), hPSCs, and their derivatives, have become a focus of research as sources of healthy mitochondria to treat tissue injury via mitochondrial transfer [168]. MSCs have been shown to transfer mitochondria to various cell types, including fibroblast, cancer, and endothelial cells [169]. Moreover, hPSC-derived MSCs have gained popularity over traditional bone-marrow-derived MSCs since they have the advantage of reduced batch-to-batch variation and high expansion ability without loss of differentiation capacity [163,170]. Human PSC-MSCs have been used for attenuating cigarette-smoke-induced damage [170] and DOX-induced cardiomyopathy [171], among others. In addition to hPSC-MSCs, other hPSC-derivatives can also act as donors of mitochondria to promote functional recovery. Human PSC-derived astrocytes have been shown to suppress dopaminergic neurodegeneration via mitochondrial transfer while mitochondria-rich extracellular vesicles from hPSC-cardiomyocytes could improve bioenergetics in mouse models of myocardial infarction [172,173].

In vivo study showed that autologously derived mitochondria injected directly into rabbits protects the heart from ischemia-reperfusion injury, characterized by decreased CK MB, cardiac troponin-I, and apoptosis [174]. Another study showed that mitochondrial transplantation in the swine heart by intracoronary delivery is a safe and effective therapy for myocardial ischemia-reperfusion injury, and improved post-ischemic function, perfusion, and infarct size [175]. A recent clinical study found that 80% of subjects (4 in 5 cases) who received autologous mitochondrial transplantation showed improvements in ventricular function, and none had arrhythmias or bleeding related to epicardial injections [176]. The group thus concluded that “Healthy autologous mitochondria harvested from nonischemic skeletal muscle can be safely injected into damaged myocardium after ischemic injury for improvement in ventricular function.”. Specifically related to DOX-induced cardiotoxicity, exogenously-transferred mitochondria in rat models of DOX-induced dilated cardiomyopathy resulted in higher LVEF, lower fibrotic area, and DNA damage in the LV myocardium compared with DOX treated rats without mitochondrial treatment [177]. Clinical trials of mitochondrial transplantation, such as those designed to ameliorate myocardial ischemia/reperfusion injury, have been registered at ClinicalTrials.gov, and the United Kingdom has passed regulations to regulate mitochondrial transfer (The Human Fertilisation and Embryology (Mitochondrial Donation) Regulations 2015 No. 572). Mitochondria transplantation provides a novel technique to protect the heart from DOX-induced damage.

### 5.5. Heart Failure Medications

Clinically, patients with DOX-induced cardiotoxicity who develop heart failure are often treated with standard heart failure medications, including angiotensin-converting enzyme (ACE) inhibitors (ACEI)/angiotensin receptor blockers (ARB), β-blockers, combined angiotensin receptor/neprilysin inhibitors (ARNI), mineralcorticoid receptor antagonists (MRA), and sodium-glucose cotransporter 2 (SGLT2) inhibitors. Although they are not specifically developed against DOX-induced cardiotoxicity, some of these medications have shown efficacy in pre-clinical models and clinical studies, both in treating heart failure after it develops and in preventing cardiotoxicity. For instance, ACEIs, such as temocapril, zofenopril, and enalapril, have been shown to prevent DOX-induced myocardial damage in animal models [178,179]. In vivo studies in rats found that the co-administration of enalapril with DOX could alleviate cardiac dysfunction induced by the latter through the preservation of mitochondrial respiratory efficiency and a reduction of free radical generation [180]. Consistent with this, a recent study involving 473 cancer patients exposed to high dose chemotherapy showed that early treatment with ACEIs, such as enalapril, could prevent the development of LV dysfunction [181]. The cardioprotective mechanisms of ACEIs against DOX are not completely clear, but may include the inhibition of the renin-angiotensin system, which plays an important role in cardiac injury and heart failure [179]. The antihypertensive properties of ACEI may help preserve cardiac performance by decreasing coronary and vascular resistance [179]. The suppression of oxidative stress and preservation of mitochondrial function may also contribute to the effects described. LCZ696, a first-in-class ARNI composed of a neprilysin inhibitor prodrug and the angiotensin receptor antagonist valsartan, could protect against DOX-induced cardiac dysfunction by suppressing mitochondrial fission, thereby improving mitochondrial respiration in rodent models [182]. β-blockers, another commonly used treatment for heart failure, have also shown promise [183]. Kawabata et al. demonstrated improved cardiac function in hearts isolated from rabbits co-treated with the β-blocker propranolol and DOX, compared with those treated with DOX alone, and this was attributed to the stabilization of myocardial mitochondria by propranolol [184]. Carvedilol has been tested in a number of clinical trials for protection against anthracyclines, and has given generally favourable responses in terms of the preservation of LVEF and other cardiac parameters [147,185,186,187]. Recent meta-analyses by Kheiri et al. and Rivera et al. both showed that the prophylactic use of carvedilol was associated with significantly superior LVEF readings among cancer patients treated with anthracyclines [188,189]. In a recent, prospective, randomized, double-blind, placebo-controlled study, carvedilol did not prevent a chemotherapy-induced reduction of LVEF, but a decrease in troponin levels and diastolic dysfunction were observed [148]. Despite such promising findings, it should be noted that carvedilol is known to have antioxidant and anti-inflammatory properties; thus, it is unclear whether the beneficial effects of carvedilol can be generalized to other β-blockers. As for SGLT2 inhibitors, Wang et al. reported that empagliflozin could ameliorate mitochondrial respiratory dysfunction in cardiomyocytes upon DOX treatment in vitro, and protected against DOX-induced cardiomyopathy in mice in vivo [190]. These effects are thought to be mediated via SIRT3 and toll-like receptor 9. MRAs (e.g., spironolactone and eplerenone) are also effective in ameliorating DOX-induced cardiotoxicity, as manifested in improved LVEF and fractional shortening, as well as reduced fibrosis and cardiomyocyte apoptosis [191,192]. Since standard heart failure medications, as described above, already have proven cardioprotective effects, repurposing them as prophylaxis against DOX-induced cardiomyopathy may be a promising therapeutic approach against this disorder.

## 6. Research Models for DOX-Induced Cardiotoxicity

**Figure 6:**
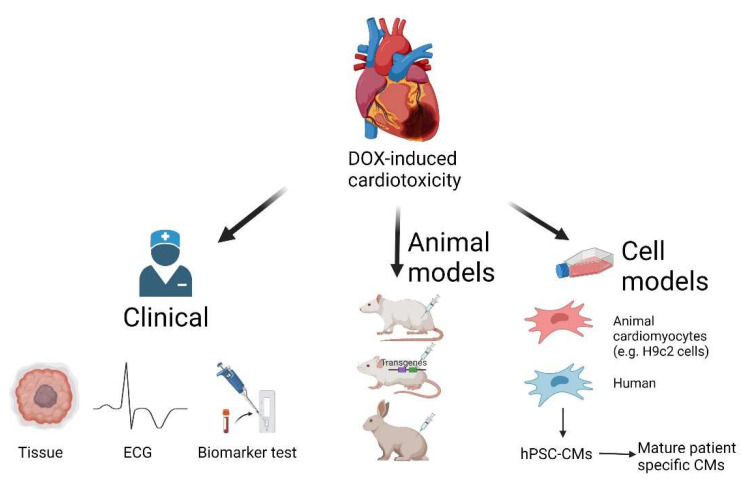
Research models for DOX-induced cardiotoxicity. Created with BioRender.com (accessed on 28 December 2021).

It has been known for decades that DOX can induce adverse effects on the heart, and much effort has been made to alleviate its cardiotoxicity. Yet, only one compound, dexrazoxane, has shown sufficient efficacy in clinical trials to be approved for use in cancer patients. Most previous studies were done using in vitro and in vivo animal models, but there has been much discrepancy between data from these models and those in clinical trials. Rodents, which are commonly used for studies of DOX, have a vastly different lifespan, cancer incidence, sensitivity to ROS, and metabolism compared to humans [150]. The rat embryonic H9c2 cardioblast cell line, and neonatal cardiomyocytes commonly used in in vitro studies (Table 1), have a different cell cycle profile and metabolism compared to adult human cardiomyocytes. Such species-specific differences might partially contribute to the conflicting results seen in animals and in patients. For these reasons, cardiomyocytes of human origin might aid in the investigations of DOX-induced cardiotoxicity (Figure 6).

Human PSCs, such as embryonic stem cells and iPSCs, self-renew and their cardiac differentiation can potentially produce an unlimited number of cardiomyocytes for research and therapy (reviewed in [193,194]). Human PSC-cardiomyocytes spontaneously contract, express cardiac-specific genes/proteins, and recapitulate key aspects of human cardiac physiology [193,194]. They are, therefore, of great value for cardiotoxicity evaluations [194,195,196].

Human PSC-cardiomyocytes have now been used in studies of DOX-induced cardiotoxicity. Many such studies, including ours, confirmed the presence of mitochondrial dysfunction and damage, in the form of increased ROS production and depolarized mitochondrial membrane potential upon DOX treatment [197,198,199]. Human PSC-cardiomyocytes can also be used to reveal the contributions of specific genes/pathways in the pathogenesis of this disorder. Examples include studies by Golforoush et al., McSweeney et al., and Li et al., which demonstrated the role of MAP4K4 and p53 in DOX-induced injury [125,200,201]. The roles of post-transcriptional regulators in DOX-induced cardiotoxicity have also been examined in hPSC-cardiomyocytes. For instance, Holmgren et al. used expression profiling to identify the proteome, transcriptome, and the regulatory miR network that might regulate DOX-induced injury [202]. The RNA-binding protein quaking was shown by Gupta et al. to inhibit DOX-induced cardiotoxicity by regulating the expression of a set of cardiac circular RNA [203]. Han et al. showed that CirclTCH, a circular RNA, can directly target miR-330-5p and protect against DOX through upregulating SIRT6, survivin, and SERCA2a [204]. Human PSC-cardiomyocytes can also facilitate biomarker discovery. Holmgren et al. reported on the differential expression of miR-34a, miR-34b, miR-187, miR-199a, miR-199b, miR-146a, miR-15b, miR-130a, miR-214, and miR-424 upon DOX treatment [205], while Chaudhari et al. revealed metabolite changes in hPSC-cardiomyocytes in response to DOX [206].

An additional advantage to the use of hiPSC technology is the ability to generate patient-derived cardiomyocytes. Somatic cells, such as blood cells or fibroblasts, can be obtained from patients who exhibit DOX-induced cardiomyopathy and reprogrammed to generate hiPSCs. Cardiomyocytes differentiated from these hiPSCs can then be used for studies of DOX-induced injury. Such a strategy was successfully employed by Burridge et al., who showed that hiPSC-cardiomyocytes recapitulate the predilection of breast cancer patients to DOX [207]. Human iPSC-cardiomyocytes derived from individuals with breast cancer who experienced DOX-induced cardiomyopathy were found to be more sensitive to DOX than those from patients who did not experience DOX-induced cardiomyopathy. The same group, again using patient specific hiPSC-cardiomyocytes, went on to demonstrate the genotype–phenotype correlation of a SNP in retinoic acid receptor-γ (RARG) and identified RARG agonist CD1350 as a potential cardioprotective treatment against DOX [208]. The use of patient-specific hiPSC-cardiomyocytes has the potential to greatly advance our understanding of DOX-induced cardiotoxicity.

Human PSC-cardiomyocytes can serve as a human in vitro model to complement in vivo animal models, but the use of these cells is not without limitations. Human PSC-cardiomyocytes are commonly considered immature, and our group and others have shown that they resemble embryonic cardiomyocytes in terms of their transcriptomic and proteomic properties [157,209,210]. Functionally, hPSC-cardiomyocytes are proliferative, with immature electrophysiological and calcium handling properties, have few mitochondria reliant on glycolysis rather than fatty acids, and are relatively resistant to oxidative damage [193,211,212,213]. The developmental immaturity of hPSC-cardiomyocytes limits their ability to fully recapitulate human adult cardiac physiology and represents a critical barrier to the use of these cells for studies of cardiotoxicity.

Much work has been done to promote the maturation of hPSC-cardiomyocytes [193,211,212,213]. They include the activation of signaling pathways and supplementation of media [209,214,215], culture on specific materials [216], formation of 3-dimentional organoids/tissue strips [217,218,219], long-term culture [220], electrical stimulation [217], and the isolation of mature hPSC-cardiomyocytes using cell surface markers [199]. We recently showed that mature hPSC-cardiomyocytes with more adult-like cardiac function can be isolated using CD36, a marker of cardiac maturation [199]. CD36^hi^ cardiomyocytes are more sensitive to injury to chemical, physiological, and drug-induced damage brought on by H_2_O_2_, hypoxia/reoxygenation, and DOX, respectively, relative to a control. Unlike conventionally grown cardiomyocytes, CD36^hi^ cardiomyocytes also recapitulate patient response to cardioprotective agents. CD36^hi^ cardiomyocytes respond to the protective effects of dexrazoxane, which is clinically proven to protect patients, but not to N-acetylcysteine, which was not successful in clinical trials. It is hoped that the use of mature, patient-specific hiPSC-cardiomyocytes will provide the impetus needed to advance the identification of therapy for DOX-induced cardiotoxicity.

## 7. Conclusions

DOX is one of the most widely used chemotherapeutic compounds for the treatment of malignancies, and has contributed to the increased survival of cancer patients. However, the cardiotoxic effects of DOX remains a key limiting factor in the use of this drug. The mechanisms of DOX-induced cardiotoxicity are complex and probably multi-factorial. In addition to established pathogenic events, such as oxidative stress and disturbed calcium homeostasis, newly discovered modes of cell damage/death, such as ferroptosis, have also been implicated in DOX-induced cardiac injury and such studies have greatly increased our understanding of DOX-induced cardiotoxicity. Great strides have also been made to expand therapeutic strategies beyond small molecule therapeutics to encompass novel treatment modalities, such as post-transcriptional regulators and mitochondrial transfer. In recent years, hPSC-cardiomyocytes have been increasingly used as a human in vitro model of DOX-induced cardiotoxicity to complete in vivo animal experiments. It is hoped that the combination of these developments can propel the study of the pathogenesis of this disorder and to identify cardioprotective strategies to protect patients against the life-threatening cardiotoxic effects of DOX.

## Figures and Tables

**Table 1:** Mitochondrial targeted cardioprotective strategies against DOX-induced cardiotoxicity.

Item	Function	Model	Reference
N-acetylcysteine	↓ Apoptosis	Neonatal rat cardiac myocytes	[80]
Alpha-tocopherol	↓ Apoptosis		
	↓ Oxidative stress		
Ascorbic acid	↓ Apoptosis		
	↓ Oxidative stress		
Dexrazoxane	↓ DNA damage	H9c2 cells	[96]
	↑ Mitochondrial membrane potential	SD rat derived ventricular myocytes	[95]
	↓ Oxidant production		
	↓ Apoptosis		
DMX-5804	↑ Cell viability	hiPSC-cardiomyocytes	[125]
	↑ Rescue spontaneous calcium cycling		
Liensinine	↑ Mitochondrial function	C57BL/6 mice, neonatal and adult mice cardiomyocytes	[126]
	↓ ROS level		
	↓ Apoptosis		
	↓ Cardiac dysfunction		
	↓ Mechanics disorder		
	↓ Ca^2+^ handling dysregulation		
	↓ Mitochondrial fragmentation		
Melatonin/metformin	↓ Apoptosis	H9c2 cells, Wistar rat	[127]
	↓ Autophagy		
	↓ ROS and inflammation		
	↑ Cell viability		
	↑ Mitochondrial function		
	↑ Mitochondrial dynamics		
	↑ Mitochondrial bioenergetics		
Dexmedetomidine	↓ Mitochondrial ROS level	C57BL/6 mice	[128]
	↓ Apoptosis		
BAY60-2770	↓ Mitochondrial membrane potential loss ↓ Caspase-3 activation	H9c2 cells	[129]
	↓ Mitochondrial ROS		
	↓ Apoptosis		
Phenylala-nine-butyramide	↑ State 3 and state 4 respiration rates	C57BL/6 mice	[130]
	↓ ROS level		
Nicotinamide riboside	↑ NAD^+^ level	C57BL/6 mice Mouse cardiomyocytes	[131]
	↑ Autolysosome clearance		
	↓ Myocardial dysfunction		
Berberine	↑ Antioxidases activities	H9c2 cells, SD rat	[50]
	↓ Malondialdehyde level		
	↓ Mitochondrial dysfunction		
	↓ Apoptosis		
miR-146a	↑ Cell viability	AC16 cells	[132]
	↑ Autophagy flux		
	↓ Apoptosis		
miR-29b	↑ Bcl-2 expression	Wistar rat, Rat cardiomyocytes	[133]
	↓ Mitochondrial membrane depolarization		
	↓ Cytochrome c release		
	↓ Caspase-3 activity		
	↓ Bax expression		
miR-378	↑ Mitochondrial membrane potential	SD rat, neonatal mice cardiomyocytes	[134]
	↓ LDH level		
	↓ Apoptosis		
miR-140-5p	↑ Morphological damage	H9c2 cells, C57BL/6 J mice	[135]
	↑ ROS level		
	↑ Creatine kinase and LDH levels		
	↓ Superoxide dismutase level		

Up arrow, increase or activate; down arrow, reduce or suppress.
